# Identification of bio-climatic determinants and potential risk areas for Kyasanur forest disease in Southern India using MaxEnt modelling approach

**DOI:** 10.1186/s12879-021-06908-9

**Published:** 2021-12-07

**Authors:** Malay Pramanik, Poonam Singh, Ramesh C. Dhiman

**Affiliations:** Environmental Epidemiology Division, ICMR-National Institute of Malaria Research, Sector 8, Dwarka, Delhi, 110077 India

**Keywords:** Bio-climatic envelope model, Kyasanur forest disease, *Haemaphysalis spinigera* tick, Monkey disease, Tick-borne disease, Hemorrhagic fever

## Abstract

**Background:**

Kyasanur forest disease (KFD), known as monkey fever, was for the first time reported in 1957 from the Shivamogga district of Karnataka. But since 2011, it has been spreading to the neighbouring state of Kerala, Goa, Maharashtra, and Tamil Nadu. The disease is transmitted to humans, monkeys and by the infected bite of ticks *Haemaphysalis spinigera*. It is known that deforestation and ecological changes are the main reasons for KFD emergence, but the bio-climatic understanding and emerging pathways remain unknown.

**Methods:**

The present study aims to understand the bio-climatic determinants of distribution of tick vector of KFD in southern India using the Maximum Entropy (MaxEnt) model. The analysis was done using 34 locations of *Haemaphysalis spinigera* occurrence and nineteen bio-climatic variables from WorldClim. Climatic variables contribution was assessed using the Jackknife test and mean AUC 0.859, indicating the model performs with very high accuracy.

**Results:**

Most influential variables affecting the spatial distribution of *Haemaphysalis spinigera* were the average temperature of the warmest quarter (bio10, contributed 32.5%), average diurnal temperature range (bio2, contributed 21%), precipitation of wettest period (bio13, contributed 17.6%), and annual precipitation (bio12, contributed 11.1%). The highest probability of *Haemaphysalis spinigera* presence was found when the mean warmest quarter temperature ranged between 25.4 and 30 °C. The risk of availability of the tick increased noticeably when the mean diurnal temperature ranged between 8 and 10 °C. The tick also preferred habitat having an annual mean temperature (bio1) between 23 and 26.2 °C, mean temperature of the driest quarter (bio9) between 20 and 28 °C, and mean temperature of the wettest quarter (bio8) between 22.5 and 25 °C.

**Conclusions:**

The results have established the relationship between bioclimatic variables and KFD tick distribution and mapped the potential areas for KFD in adjacent areas wherein surveillance for the disease is warranted for early preparedness before the occurrence of outbreaks etc. The modelling approach helps link bio-climatic variables with the present and predicted distribution of *Haemaphysalis spinigera* tick.

**Supplementary Information:**

The online version contains supplementary material available at 10.1186/s12879-021-06908-9.

## Introduction

Kyasanur forest disease (KFD) is a zoonotic tick-borne viral disease, first reported from the forested area of Shivamogga district, Karnataka, in 1957 [1]. The disease is caused by the KFD virus belonging to the family *Flaviviridae* and genus *Flavivirus*, measuring about 40–60 nm in diameter [2–4]. The KFD virus genome consists of 10,774 nucleotides of single-stranded, positive-sense RNA encoding a single polyprotein [3]. The virus genome is very similar to that of Alkhurma Hemorrhagic Fever Virus (> 92% homologous), which is primarily found in Saudi Arabia [4]. These two species both belong to the family *Flaviviridae* and diverged over 700 years ago and have thus remained geographically separated [4].

The virus was found to be highly infectious, as evidenced by several infections in laboratory and field staff [5, 6], which resulted in work suspension until a proper Biosafety Level-3 laboratory was established at ICMR-National Institute of Virology in 2004. In nature, the virus is found in ticks, monkeys, shrews, bats and small mammals [7, 8]. KFD is transmitted to animals and humans by the infected tick bites, mainly *Haemaphysalis* *spinigera* [9–12]. The incubation period of the KFD virus is ~ 3–8 days [13]. The symptoms of KFD include a high fever with frontal headaches, severe muscle pain, vomiting, chills, and other gastrointestinal symptoms [13]. Bleeding problems can occur 3–4 days after the onset of the initial symptom. Patients may have unusually low blood pressure as well as low red and white blood cell, and platelet counts [13].

A variety of animals are thought to be reservoir hosts for the disease, including rats, porcupines squirrels, shrews, and mice. Monkeys are the main amplifying host of KFD, they come out to human dwellings and their death signals the outbreak in human beings [7, 14]. Monkeys (red-faced *Macaca radiata* and black-faced *Presbytis entellus*) are the reservoirs of KFD, but they also die due to KFD [8]. The KFD virus is highly contagious in the *bonnet macaques* and *surili Presbytis entellus.* They become extremely virulent and infect ticks. *Haemaphysalis spinigera*, a forest tick, serves as the disease’s vector [11]. The bite of tick nymphs causes infection in humans. Because the human domestic environment does not support ticks, man is a terminal host, and there is no human-to-human transmission [9, 12].

**Fig. 1:**
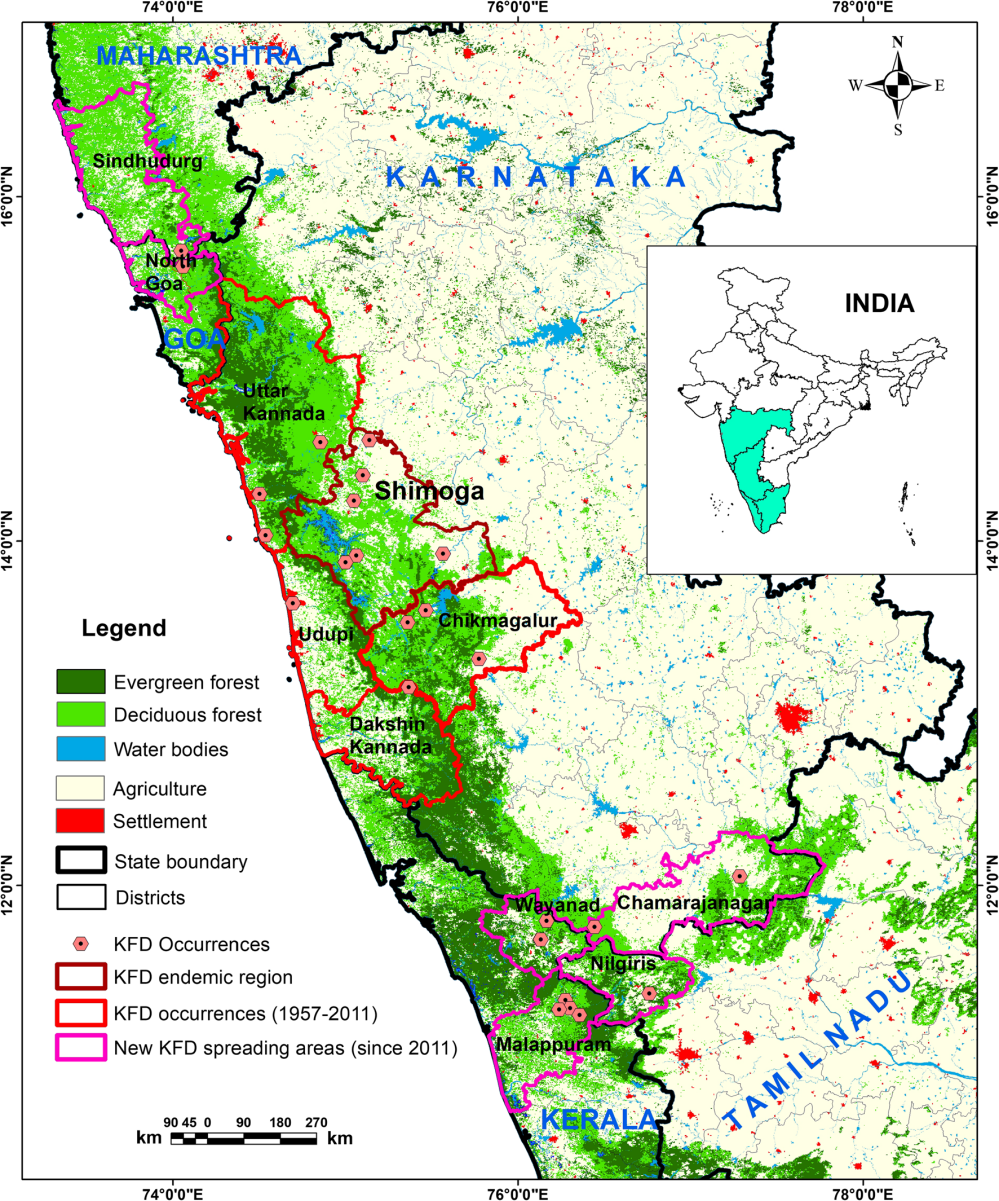
Locations of reported distribution of *Haemaphysalis spinigera* tick, KFD endemic areas till 2011 and afterwards

It frequently occurs in semi-evergreen, evergreen, deciduous and moist forests in southern India only [14] and has also been related to developmental activities resulting in deforestation [15] and ecological changes [8]. Population with occupational exposure to outdoor or rural settings (i.e., herders, hunters, farmers, and forest workers) in the villages are potentially at risk of the disease if they contact infected *Haemaphysalis spinigera* ticks [3, 8]. The disease prevails in the dry months from November to May, when the nymphs’ density is maximum in the forest due to favourable moisture in the soil of forest areas [16–18]. The average temperature in the dry months has increased substantially over the years and this has led to water crisis in the region [15]. The disease is localized in several districts, namely Chikmagalur, Shivamogga, Udupi, Dakshina Kannada, and Uttar Kannada of the Karnataka state, India [6, 12]. Since the first reporting of the disease from the Shivamogga district in 1957, several sporadic cases and outbreaks have been reported every year in the same region [4, 7, 19, 20]. But, in the past few years (i.e., 2013 onwards), the geographical range of the disease has extended to the districts in Kerala, Goa, Maharashtra, and Tamil Nadu (Fig. 1).

The reason for the spread of KFD to nearby areas is not known. It is assumed that ecology and bioclimatic variables are responding to such spatial distribution. Previous studies by Ajesh et al. [14], Banerjee and Bhatt [5] showed that the disease extends, ranges and changes happen due to the influence of ecological destructions and by the eventual effects of resulting climate change [5, 14]. But, the climatic factors are not fully understood. Therefore, to prove this hypothesis, we have used Ecological Niche to establish the climatic predictors of tick distribution in the study region based on presence/absence data [24, 25]. The model has been devised by Phillips et al. [24] and Yang et al. [25], based on the MaxEnt entropy algorithm [24] can evaluate the predictors role as well as predict the distribution of a species, even with the very limited presence data [26, 29]. It is a well-established algorithm to identify the potential suitability of different epidemic diseases, vectors, and fauna & flora species [26–30]. The risk assessments and prediction of hosts and vectors using the MaxEnt algorithm have been investigated in other vector-borne diseases like malaria, leishmaniasis, Rift Valley fever virus, dengue, West Nile virus, and japanese encephalitis [31–35]. The results of the species distribution model can help improve tick monitoring, surveillance and guide for implementing control programmes [31, 36, 37].

It was thought prudent to understand the bioclimatic factors responsible for present distribution as well as the potential distribution of *Haemaphysalis spinigera* in India. Therefore, the present study used the ecological niche modelling (MaxEnt) approach to determine the risk areas of KFD and climatic sensitivity of *Haemaphysalis spinigera* for southern India based on field survey and existing occurrence data. In addition, our modelling results explained the link between model-based favourable climatic conditions and the possibility of monkey death and KFD tick expansion in the endemic and potential areas in other parts of India. The tool is the only efficient strategy for controlling and preventing the disease to find out the biological and climatic risk of *Haemaphysalis spinigera* and KFD.

## Materials and methods

### *Haemaphysalis spinigera* tick occurrence data

Data on reported availability of *Haemaphysalis spinigera* were collated by systematic and comprehensive literature retrieval from the google scholar, Cochrane library, PubMed and the Web of science database, by using the keywords Kyasanur forest disease, *Haemaphysalis spinigera* occurrence, monkey death by KFD virus, KFD in India, human cases of KFD (Additional file 1). The literature dealing with the availability of *Haemaphysalis spinigera*, the occurrence of KFD cases or monkey death from 1957 onwards were considered to ascertain the coordinates. Location of confirmed cases (human cases and monkey death) were converted into point features (exact latitude and longitude, 1 km × 1 km) or polygon features (i.e., localities, villages, districts) georeferenced using Google Earth and Arc-GIS for the rectification of latitude and longitude [38]. When the name of the locality or village could not be identified at the administrative level, the coordinates were overlaid in a geographic information system (GIS) and assigned to the appropriate polygon feature [38]. All the locations of the occurrence of *Haemaphysalis spinigera* tick were transformed into WGS 84 datum using Arc-GIS software. Since the present study was conducted by the resolution of 30 arc-second (approximately 1 km × 1 km resolution), localities within one pixel were selected as one occurrence point. Altogether, 34 locations with confirmed human cases, monkey deaths or availability of *Haemaphysalis spinigera* ticks were georeferenced from all the reported areas of Karnataka, Maharashtra, Kerala, Goa, and Tamil Nadu (Fig. 1) (Additional file 1: Table S1). These occurrence locations were used for the MaxEnt model input, as the model predicts very accurately with very limited presence and absence datasets [26, 29, 36].

### Bio-climatic variables

Bio-climatic variables are biologically more significant to identifying plants and animals’ physio-ecological resistance than simple temperature and rainfall [39, 40]. Therefore, these variables are commonly used in bio-climate envelope modelling [31, 41]. The study used 19 bio-climatic variables as potential predictors of *Haemaphysalis spinigera* distribution (as shown in Additional file 1: Table S2). Raster-based bio-climatic variables were collected from the WorldClim Version2 (http://www.worldclim.com/version2). The spatial resolution of these bio-climatic layers is ∼ 1 km (30 arc seconds) and show extremity and seasonality of temperature, annual trends of precipitation and temperature parameters.

Of 19 bio-climatic variables, five extremely correlated variables, having a negligible effect on the model, were removed to reduce the masking effect and produce a model with better predictability [42]. The test was run by Pearson’s correlation coefficient (r) using ENM Tool (version 1.3), and a cross-correlation ‘r’ value of more than 0.80 was taken as a cut of threshold [25, 42] (Additional file 1: Table S3). Finally, the remaining 14 bio-climatic variables with a higher permutation significance and percent contribution were used for modelling. Based on the MaxEnt produced response curves, the relationship between bioclimatic variables and habitat suitability for *Haemaphysalis spinigera* occurrence were evaluated.

### Predictive modelling

The ArcGIS 10.3 and ENVI 5.1 softwares were used to generate raster-based spatial layers of the bio-climatic variables. The maximum entropy (MaxEnt) modelling is a machine learning algorithm [24] that calculates the probability distribution for a vector or species location based on different environmental restraints. The model executes well even with fewer sampling points than other machine learning methods [43]. Using presence-only vector/species location points to predict the potential distribution based on MaxEnt theory [24]. The basic principle of this algorithm is to ensure that approximation meets any limitations on the unknown points, meaning that the calculated probability of unknown distribution represents less number of constraints with a set of extra choices [44, 45]. However, in this study, we used 34 locations’ data about the presence of *Haemaphysalis spinigera* and generated pseudo-absences. The maximum entropy algorithm randomly selected about 10,000 background points. Data on the presence of ticks were divided into 75% random samples to calibrate the model, and the 25% random samples were utilized to assess the model performance. We used the subsampling method to create a stable model because it has advantages over bootstrap and cross-validation [46, 47], and 50 replicates were chosen to run the model.

The model also suggests settings to assess the complexity of the model by altering regularization multipliers and feature classes. Sixteen different combinations of the feature classes were created to identify the appropriate feature by retaining the linear function in each feature, then used for model performance. In order to balance the fit of the model and avoid overfitting, regularization multipliers were used [48]. The selected default value for model calibration is 1.0. In total, 123 models combinations were created for choosing the best fit model in different settings. Other values of the model were set as default to get better results.

### Threshold identification

For model results indicating the probability of presence (suitability of a species), the logistic value ranging from 0 (unsuitable) to 1 (max. probability of presence) was used [24]. By applying ‘max SSS’ (maximum test sensitivity with specificity) logistic threshold value, binary unsuitable/maximum suitable map has been prepared. Specificity (Sp) and Sensitivity (Se), which are independent, implies the likelihood of a model that adequately forecasts a species absence and presence in any location and measures the commission and omission errors. Sp and Se are distincts and not influenced by predominant across models [49]. In the ROC curve, the ‘max SSS” identifies a point in which the tangent slope is 1 that demonstrates 1-specificity and sensitivity for maximizing TSS value. The value can be utilized as an efficient threshold value when the only occurrence or target species presence data are available and used extensively [50–52]. This binary raster was used to show the potential distribution of the *Haemaphysalis spinigera* ticks using SDM toolbox 2.0. A bias file has corrected the selection of backgrounds for latitudinal changes resulting from the geographical coordinate systems [53].

### Model assessment and validation

**Fig. 2:**
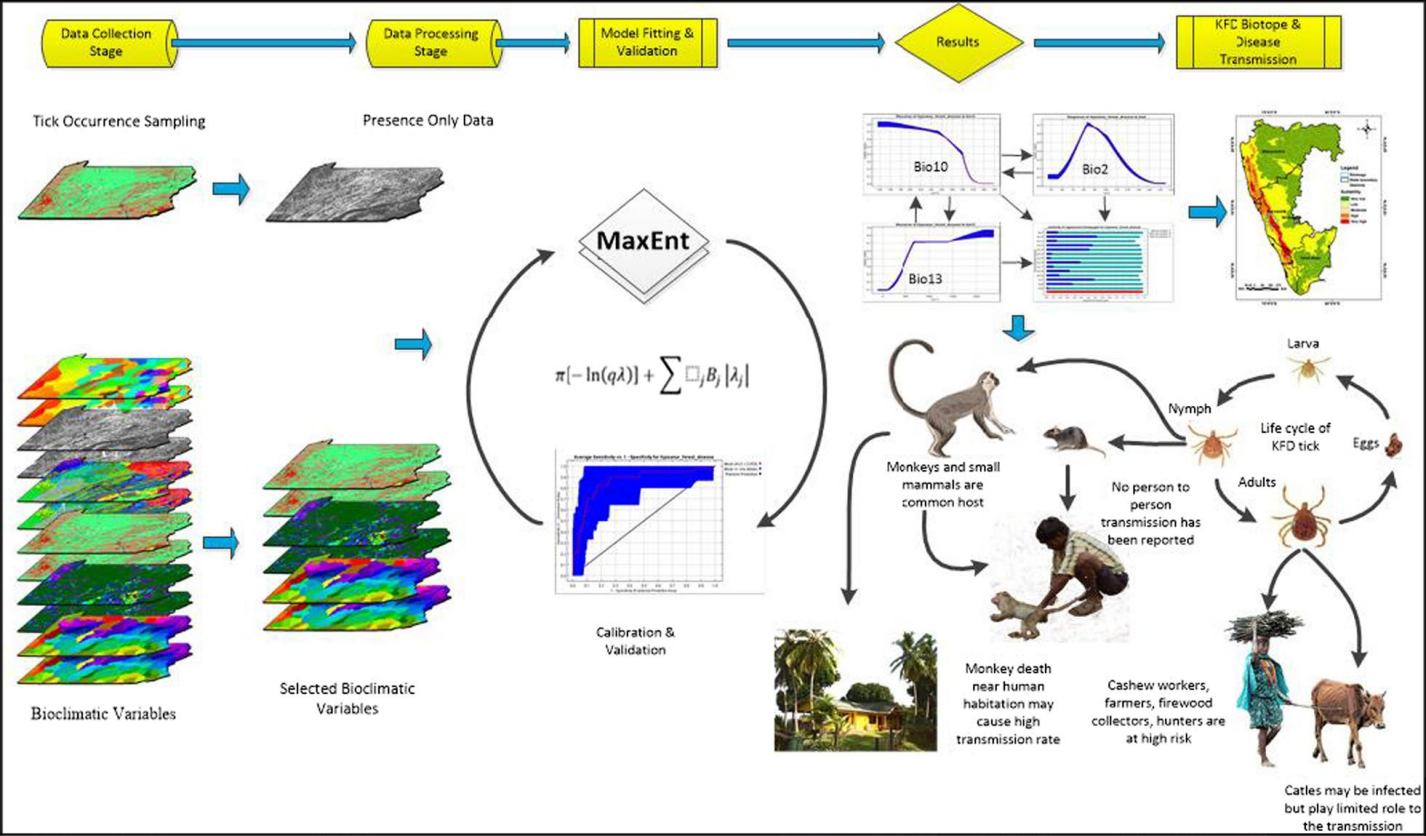
Methodological flow diagram showing the link between suitable climatic conditions and disease transmission of Kyasanur forest disease

To estimate the goodness of fit of the model, the Area Under the receiver operating characteristics Curve (AUC) was used, and the highest value was indicated as the best performer. The AUC is a threshold-independent technique of a model assessment to discriminate outcomes of presence/absence [54]. AUC values vary from least value 0 to the highest value 1. The 0.5 value signifies that the model findings were less than random, while the 1.0 value indicates complete discrimination [54, 55]. In the Jackknife test, the contribution of the bio-climatic factors was also measured. The detailed methodological flow diagram in this work is shown in Fig. 2.

## Results

### Model performance

**Fig. 3:**
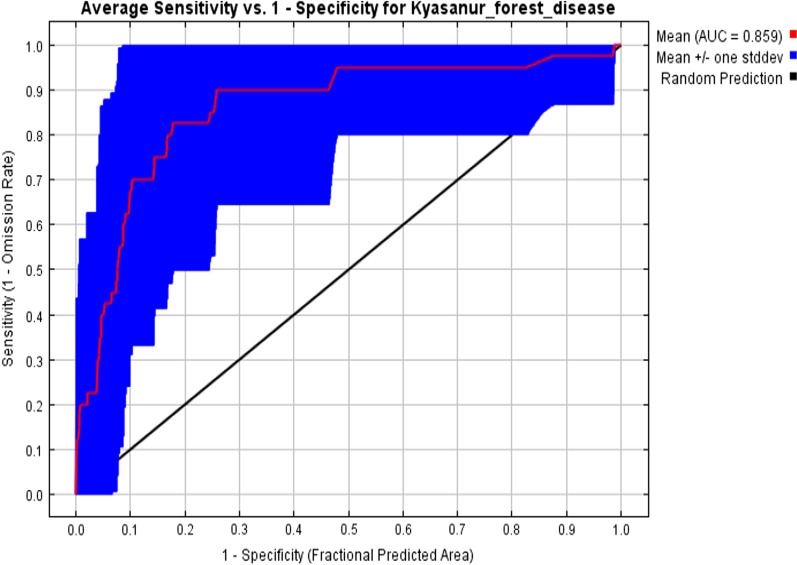
The ROC curve for *Haemaphysalis spinigera* tick showing different AUC values

The logistic results for the presence of tick suitability and the distribution of Kyasanur forest disease were found highly significant. The AUC results for the training sample are 0.898, and for the test, the sample is 0.859 (Fig. 3). This suggests that the bio-climatic variables set, used for the prediction model, and interpreted the predicted potential suitability very well with very high accuracy. The optimum threshold value, which provides equal weight to specificity and sensitivity, was selected to classify suitable areas of *Haemaphysalis spinigera*.

### Identified bio-climatic variables for distribution of *Haemaphysalis spinigera*

**Fig. 4:**
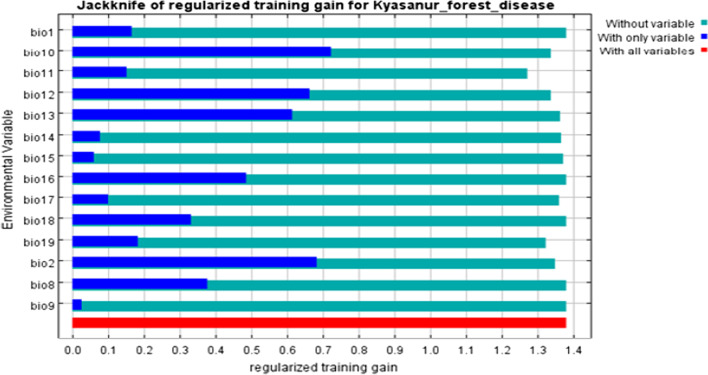
The Jackknife test results indicating the relative importance of bio-climatic variables

Of 14 bio-climatic variables used for modelling, more influential variables affecting the spatial distribution of *Haemaphysalis spinigera* were the average temperature of the warmest quarter (bio10, contributed 32.5%), average diurnal temperature range (bio2, contributed 21%), precipitation of wettest period (bio13, contributed 17.6%), and annual precipitation (bio12, contributed 11.1%). The cumulative contribution of these variables was 82.2%. The variable having high permutation importance was the average temperature of the warmest quarter (40.1%). The remaining 12 variables, i.e., annual mean temperature (Bio1), average temperature of the driest quarter (bio9), average temperature of the coldest quarter (bio11), rainfall of the warmest quarter (bio18), mean temperature of the wettest quarter (bio8), precipitation of wettest quarter (bio16), rainfall of driest quarter (bio17), precipitation of driest period (bio14), and precipitation seasonality (bio15) contributed 17.8% altogether to the suitability model (Table 1). Therefore, the average temperature of the warmest quarter and mean diurnal temperature change are very significant variables contributing to the risk area mapping of KFD. Both variables generate the best prediction results when used individually from Jackknife analysis. Figure 4 shows the Jackknife test results of the climatic variable importance as estimated by the model.

**Table 1:** Selected set of bio-climatic variables after PCA results and their contribution to the KFD suitability

Id of bioclimatic variable	Selected bio-climatic variable	Contribution (%)	Optimum bio-climatic conditions
Bio1	Annual mean temperature	0.1	23–26.2 °C
Bio2	Mean diurnal temperature range	21	8–10 °C
Bio8	Mean temperature of wettest quarter	2.4	22.5–25 °C
Bio9	Mean temperature of driest quarter	0.5	20–28 °C
Bio10	Mean temperature of warmest quarter	32.5	25.4–30 °C
Bio11	Mean temperature of coldest quarter	3.9	16.5°–24 C
Bio12	Annual precipitation	11.1	> 1400 mm
Bio13	Precipitation of wettest period	17.6	500–650 mm

### Association of *Haemaphysalis spinigera* tick to climatic variables

**Fig. 5:**
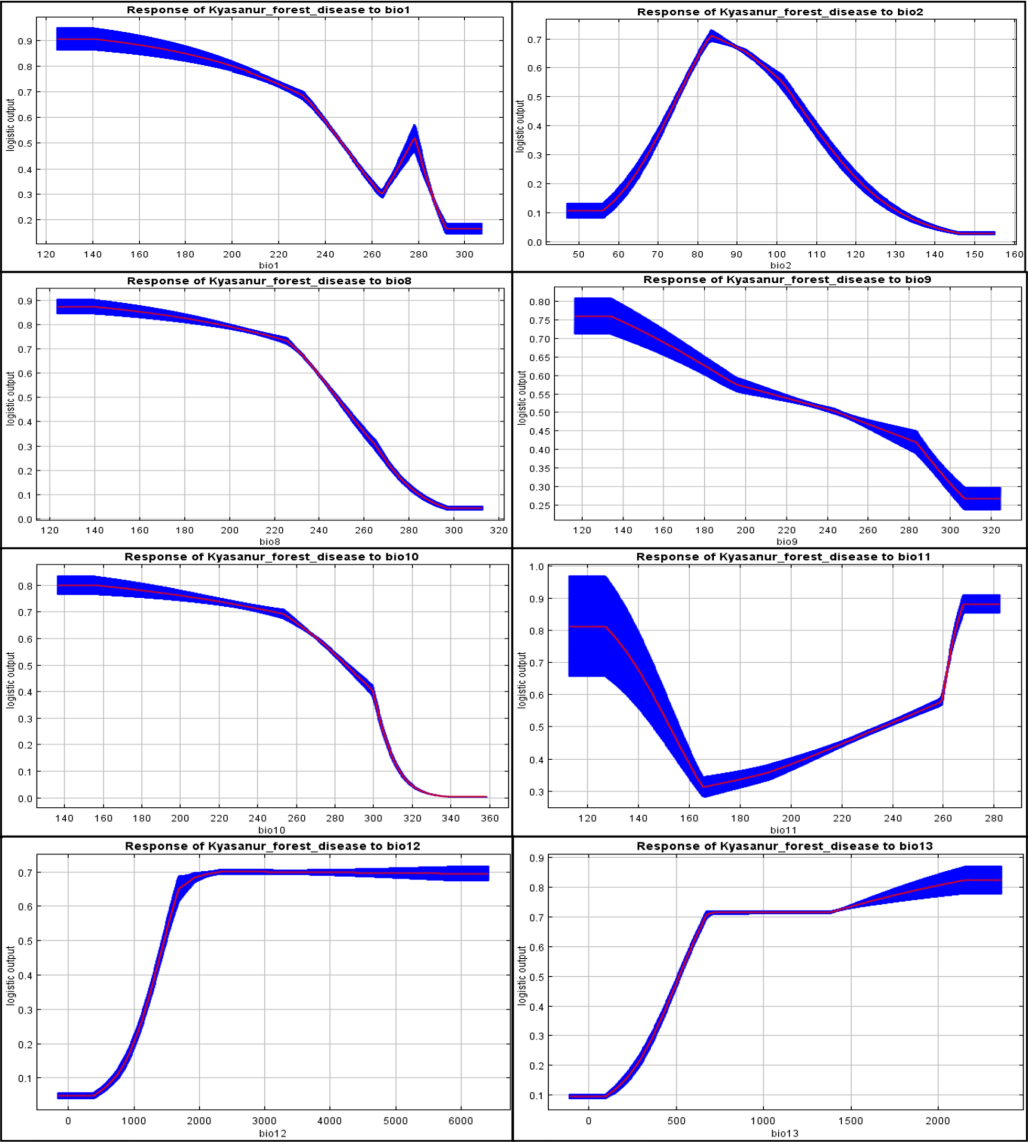

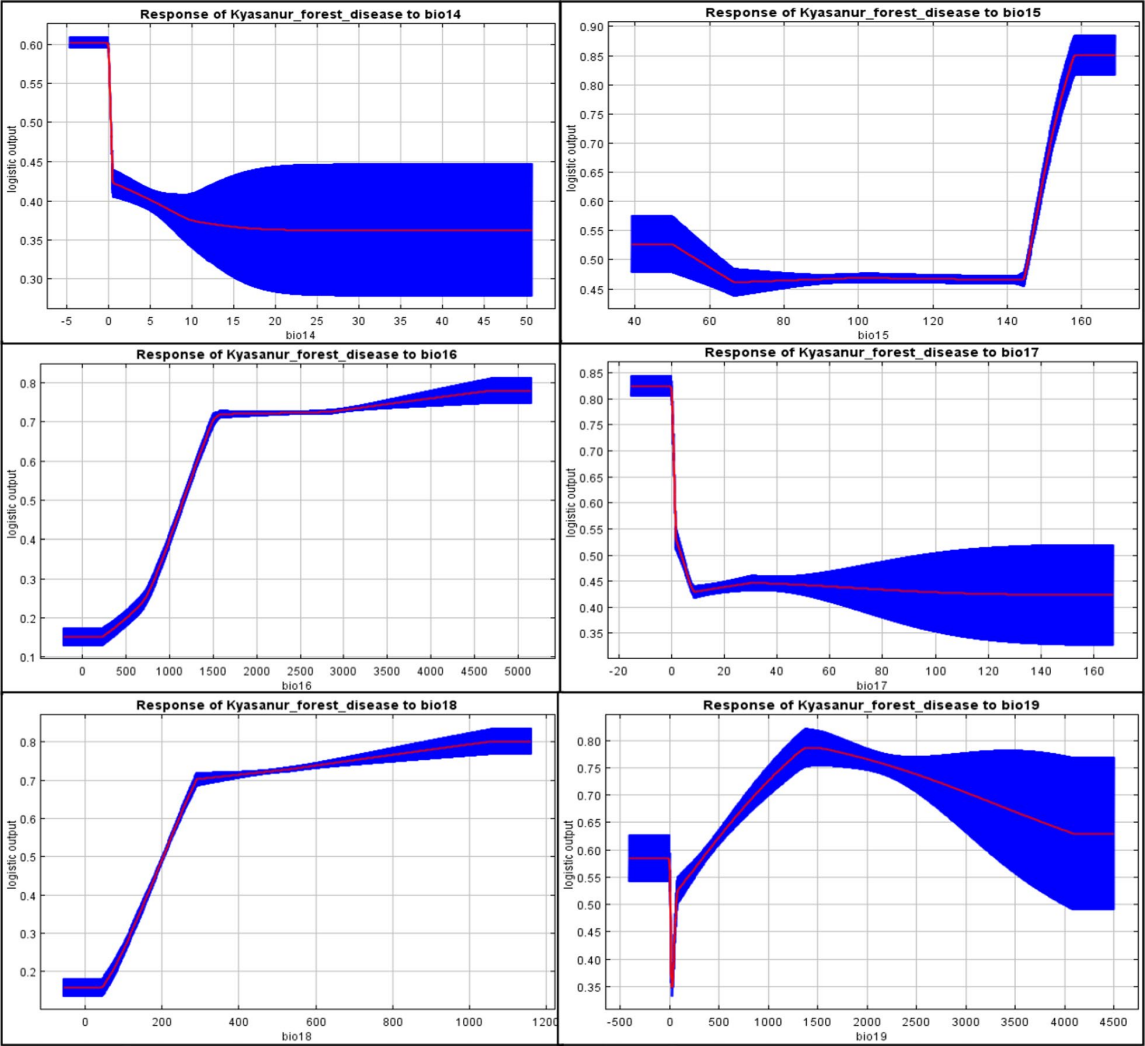
Relationship between selected climatic variables and probability of the presence of *Haemaphysalis spinigera* ticks. **a** Annual mean temperature (bio1, °C), **b** mean diurnal temperature range (bio2, °C), **c** mean temperature of wettest quarter (bio8, °C), **d** mean temperature of driest quarter (bio9, mm), **e** mean temperature of warmest quarter (bio10, mm), **f** mean temperature of coldest quarter (bio11, CV), **g** annual precipitation (bio12, mm), **h** precipitation of wettest period (bio13, mm), **i** precipitation of driest month (bio14, mm), **j** precipitation seasonality (bio15, CV), **k** precipitation of wettest quarter (bio16, mm), **l** precipitation of driest quarter (bio17, mm), **m** precipitation of warmest quarter (bio18, mm), **n** precipitation of coldest quarter (bio19, mm). Red curves indicate the average response and blue margins signify ± SD estimated over 50 replicates

Figure 5a–n indicates individual response curves of the association between each bio-climatic variable and the possibility of *Haemaphysalis spinigera* tick presence as estimated by the model. The response curves from the model’s performance show the differences in the logistic value conveyed by alteration in each parameter if the mean value of all other variables is preserved. However, there is an overall non-linear negative relationship detected for the annual average temperature (bio1), the average temperature of the warmest quarter (bio10), the average temperature of the driest quarter (bio9), and mean temperature of the wettest quarter (bio8) indicating that higher the temperature intensity, the lower would be the *Haemaphysalis spinigera* tick distribution. *Haemaphysalis spinigera* tick preferred habitat having an annual mean temperature (bio1) between 23 and 26.2 °C, mean temperature of the driest quarter (bio9) between 20 and 28 °C, and mean temperature of the wettest quarter (bio8) between 22.5 and 25 °C. Precipitation of the wettest period and annual precipitation showed a non-linear positive response, indicating that the higher the precipitation intensity, the higher the *Haemaphysalis spinigera* tick distribution. The mean temperature of the warmest quarter (bio10) represented the temperature in the warmest season and revealed a significant probability of *Haemaphysalis spinigera* presence between 25.4 and 30 °C. The mean diurnal temperature range is the difference between daily maximum and daily minimum temperature, and it revealed a very high probability of tick presence between 8 and 10 °C. The response to precipitation of the wettest period (bio13) showed that precipitation of 500–650 mm highly favoured the presence of *Haemaphysalis spinigera* tick. The other optimum bio-climatic parameters for *Haemaphysalis spinigera* tick suitability are shown in Table 1. Subsequently, the high tick population could be a cause for monkey death and the human case.

### Potential risk areas of *Haemaphysalis spinigera*

**Fig. 6:**
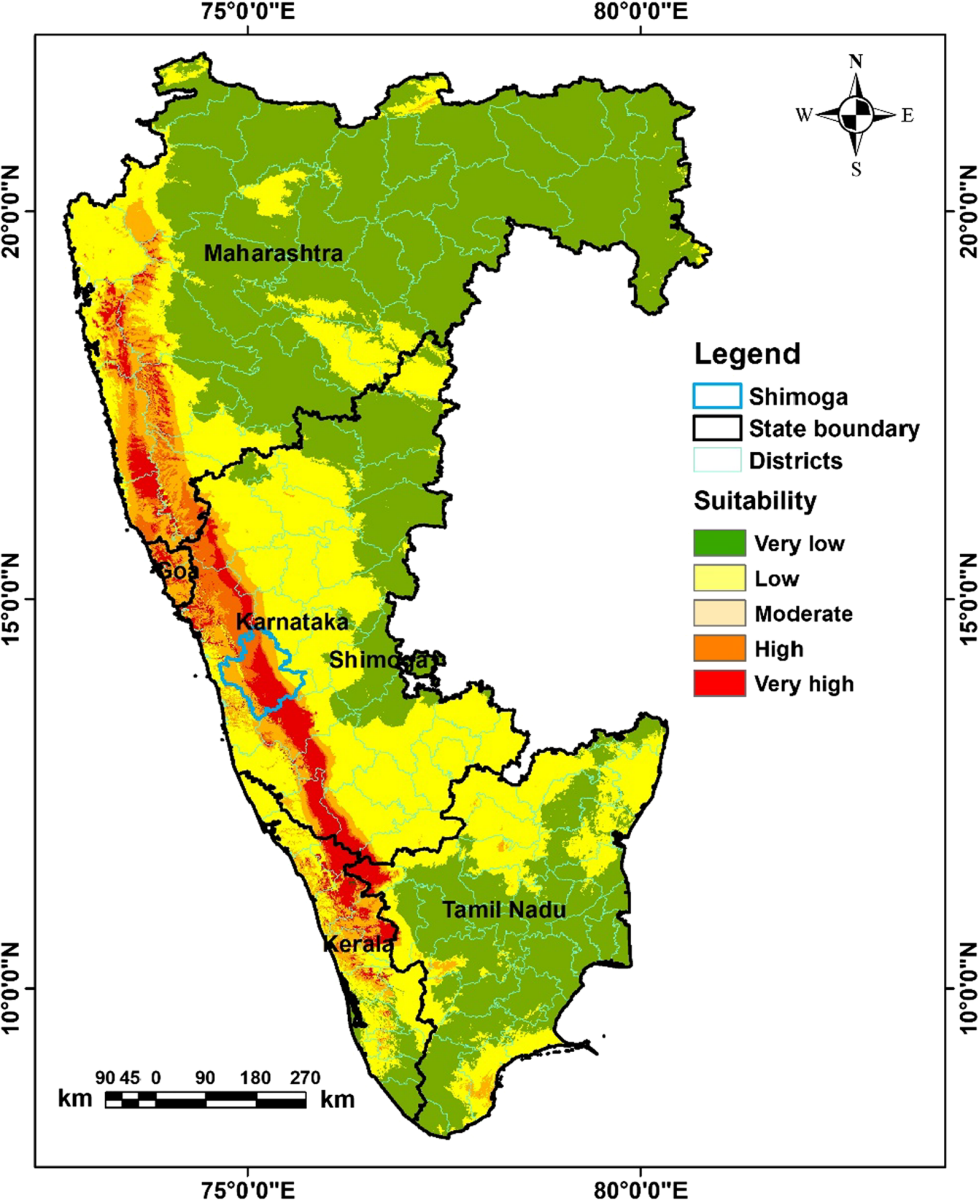
Map of the predicted potential distribution of *Haemaphysalis spinigera* tick

We converted the predicted probability map of *Haemaphysalis spinigera tick* suitability from the MaxEnt model to presence and absence using the ‘max SSS’ logistic threshold value. The predicted presence areas were classified as very high to moderately suitable areas, and the absence areas were classified as non-suitable areas for *Haemaphysalis spinigera*. Based on the proportion of bioclimatic suitability areas, the potential suitability map was classified into five different suitability categories, i.e., ‘very high suitability’ (0.80–1.0), ‘high suitability’ (0.79–0.60), ‘moderate suitability’ (0.59–0.40), ‘low suitability’ (0.39–0.20), and ‘very low suitable’ class (0–0.19). The predicted potential areas of *Haemaphysalis spinigera* under present bio-climatic settings are shown in Fig. 6. As per results, about 26,429 km^2^ (4%) area comes under ‘very high potential’ for *Haemaphysalis spinigera*, followed by ‘high potential’ at 18,258 (3%) and ‘moderate potential’ at 45,759 km^2^ (7%) (Table 2). The results further show that Karnataka is the most potential region, followed by Maharashtra, Kerala, Goa, and Tamil Nadu. The high to very high suitable areas are Shivamogga, Chamrajnagar, Chikmagalur in Karnataka; Kozhikode and Wayanad districts in Kerala; Raigad, Ratnagiri districts in Maharashtra; Nilgiris district in Tamil Nadu; North Goa district in Goa, requiring continuous vigilance (Fig. 6). The district-wise suitability map shows linear spatial clustering along the western Ghats as a very high-risk zone of the potential distribution of *Haemaphysalis spinigera*. In those districts, extensive survey, continuous vigilance is required, especially from November to March, when most KFD cases occur. The percentage of the risk area in each state is shown in Table 3.

**Table 2:** Area under different risk classes for KFD tick

Risk categories	Area (sq. km)	Area in %
Very low risk	359,767	53
Low risk	220,650	33
Medium risk	45,769	7
High risk	18,258	3
Very high risk	26,429	4
Total	670,873	100

**Table 3:** State-wise high risk area for KFD tick

States	High suitable area (km^2^)	% of very highly suitable area in the total suitability	% of very highly suitable area in the state
Goa	2056	5.18	55.66
Karnataka	23,265	58.66	12.16
Kerala	4765	12.02	12.64
Maharashtra	7198	18.15	2.34
Tamil Nadu	2376	5.99	1.82
Total area	39,660	100	

## Discussion

This is the first study to link the bioclimatic variables with the potential distribution of *Haemaphysalis spinigera* tick in South India. Based on tick occurrence records, MaxEnt modelled the potential distribution of *Haemaphysalis spinigera* tick as 23,265 sq. km (58.66%) spread over the Shivamogga, Chamrajnagar, and Chikmagalur districts in Karnataka; 4765 sq. km (12.02%) in Kozhikode and Wayanad districts of Kerala; 7198 sq. km (18.15%) in Raigad, Ratnagiri districts of Maharashtra; the Nilgiris districts in Tamil Nadu; North Goa district in Goa (Fig. 6). The region is characterized by the mix of wet and dry climatic conditions, but most of the KFD affected regions are located in the eastern regions of the Western Ghats. These locations lie in the rain shadow region, receive far less rainfall and long dry spells [36]. Therefore, most of the districts in Karnataka as well as southern India are suitable for KFD in present climatic conditions as they are located in the rain shadow [36].

The relationship between the growing distribution of *Haemaphysalis spinigera* tick and variations in the mean temperature of warmest quarter (bio10) and mean diurnal temperature range (bio2) means dry and warmer climatic conditions were evident in the model results [16–18]. The risk areas of *Haemaphysalis spinigera* tick were more intense in the locations with precipitation of wettest period 500–650 mm, mean diurnal temperature range 8–10 °C and mean temperature of the warmest quarter 25.4–30 °C, which is relatively hot and dry over the study region. KFD’s transmission occurs during the non-rainy season as the nymphs of *Haemaphysalis spinigera* ticks are active during this season (Fig. 2)*.*

Human encroachment and deforestation in the affected area increases the encounters with infected *Haemaphysalis spinigera* ticks. Furthermore, deforestation affects the local precipitation pattern, thus impacting the micro-climate of the region. The results shows that the predicted risk areas are expanding in hot and drier climatic conditions, as the tick’s distribution and lifecycle heavily depend on the precipitation and temperature of this region [15]. Human activity peaks post monsoon for the paddy harvest, gathering firewood, forest products and the collection of other livelihoods [8, 15]. The expansion in areas of the tick population in Africa was related to variations in temperature and precipitation [56]. Moreover, a warmer temperature has been found as the most influential factor for the geographic range shifting of some tick species [57, 58]. According to the previous studies, climatic variables have contributed to the expanded range of ticks, potentially increasing the risk of Lyme disease e.g. in areas of Canada where ticks were previously unable to survive [59]. Temperature has a significant impact on the life cycle and prevalence of deer ticks [60].

**Fig. 7:**
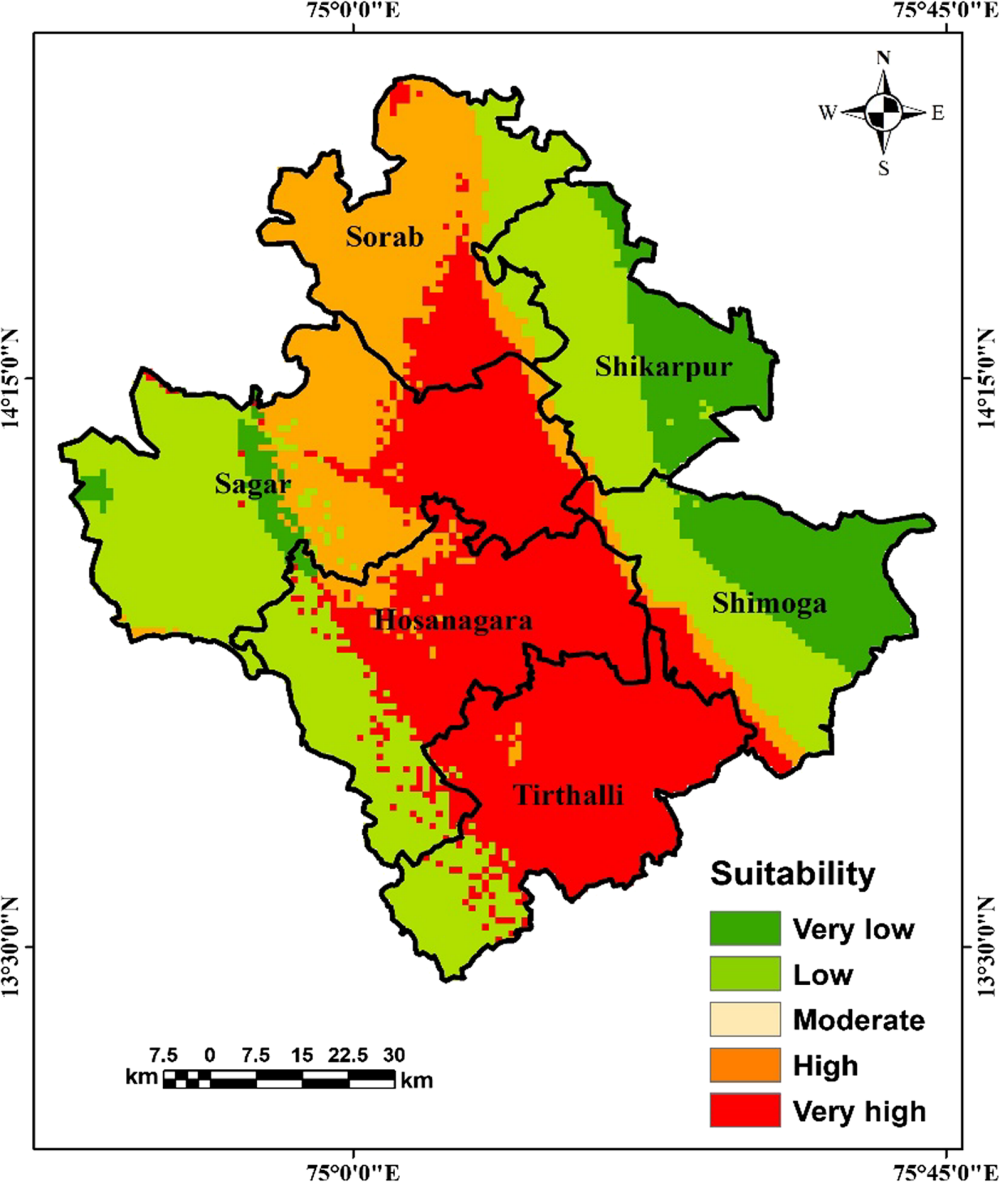
Predicted potential distribution of *Haemaphysalis spinigera* tick in endemic region of Shivamogga district

In the states of Karnataka, Goa, Maharashtra, Kerala, Tamil Nadu, the increasing distribution of *Haemaphysalis spinigera* tick mainly was seen in the deciduous and neighbouring semi-evergreen and evergreen areas [61] (Fig. 1). Studies showed that these forested lands were more prone to dry climate with decreasing precipitation [62]. The decrease in precipitation during the pre-monsoon (southwest) resulted in short-term meteorological droughts in this region [63, 64], which increases the suitable areas of *Haemaphysalis spinigera* tick. Secondly in the areas with reported KFD cases, annual rainfall was relatively lower, and temperature somewhat higher than KFD cases free areas. Human get infected through the bite of nymphal ticks, which are mostly active during the hot and dry season in the study region [62]. According to Raj and Azeez [65], and Nair et al. [66] the aridity index increases significantly in the region, therefore the risk areas of *Haemaphysalis spinigera* tick may influence the significant rise in KFD cases in the region. The results of the present study indicates that the potential tick suitability increases, when the mean temperature of the warmest quarter ranges between 25.4 and 30 °C, and more than 30 °C is not suitable. Even ticks die because of water loss due to destruction in the integument. Moreover, the endemic areas of KFD in the Shivamogga district are also shifting from Shivamogga to Thirthahalli, Hosangara taluka, which are presently the most endemic region in Shivamogga district (Fig. 7). According to bio-climatic data, a suitable diurnal temperature range and mean warmest month temperature with low precipitation was found in the high endemic talukas, as mentioned earlier than the low endemic areas of Shivamogga taluka which can explain the reason for the high endemicity of KFD (Fig. 2).

Accordingly, our results also indicate a significant increase in distribution of *Haemaphysalis spinigera* or risk areas of KFD with favourable climatic conditions (warmest month temperature and diurnal temperature range) in seven regions, namely Shivamogga, Chamarajanagar, Bandipur National Park, Madurai Tiger Reserve, Wayanad, Sattari, Malappuram, where the highest number of monkey death and human cases were observed.

Moreover socio-economic factors may also be having an influence on KFD transmission in Shivamogga, which need further study. Other factors like, migration of ticks and reservoir hosts, increased human contact with infected animals, migratory birds and bats including the climate change may play an important role to understand the threat of KFD cases.

Species distribution modelling is known as ‘habitat suitability, ‘ecological niche’, and ‘potential distribution’ modelling and these are used to predict the suitable habitats [67] of a tick species. In this study, we limited the increasing distribution of *Haemaphysalis spinigera* tick potentiality in terms of climatic variables and tick occurrences’ to identify the climatic determinants in the habitat alteration in southern India. The role of deforestation has been found in hot spots of KFD in western Ghats [68] which may further be elucidated using satellite remote sensing.

We omitted land use and land cover variables because the ticks’ distribution is associated with deciduous, evergreen, and semi-evergreen forest [61]. The role of low mammalian richness has also been found in outbreaks of KFD [69].

Soil characteristics were also excluded from this study since the high resolution and more accurate data were not available. Moreover, we also excluded the role of host-agent-environment factors due to the lack of proper information over this region. Regardless, further spread of *Haemaphysalis spinigera* tick may be affected by climate change, emphasizing the need for further studies.

It has been observed that the improvement in diagnostic assay through Virus Research Diagnostic Lab established in Shivamogga and tick monitoring are affecting disease awareness and better reporting.

Our study used Maxent bio-climatic model and studying the ecology was not the main aim. However, detailed ecological studies using remote sensing, forestry, anthropology and veterinary domains for threat of KFD are warranted. Understanding the ecology and epidemiology of a tick-borne disease is indeed multifactorial which cannot be answered by one study. That is why One Health approach is being adopted to address such zoonotic diseases comprehensively.

## Conclusion

The bio-climate envelop modelling approach has been found as a useful tool to link bio-climatic variables with the present and predicted distribution of Kyasanur forest disease. It has predicted the potential climatic suitability of KFD in Shivamogga, Chamrajnagar, Chikmagalur in Karnataka; Kozhikode and Wayanad districts in Kerala; Raigad, Ratnagiri districts in Maharashtra; the Nilgiris district in Tamil Nadu; North Goa district in Goa. These districts are categorized as dry and hot climates than other districts of the study area. The predicted potential risk map emphasized the significance of climatic variables in identifying the potential risk district for KFD warranting surveillance for KFD in hitherto KFD-free contiguous areas. Better understanding of KFD emergence linking with climatic factors with ecology using satellite remote sensing, deforestation and mammalian population density will help to build accurate surveillance system allowing to track spread and emerging pathways. Future studies should be designed incorporating further risk variables (i.e. monkey dispersal pattern, seasonal forest characteristics, climate change, socio-economic factors host-agent-environment factors (One Health approach) in Shivamogga district, western Ghats and the southern states of India.

## Supplementary Information

**Additional file 1: Table S1.** Showing reported tick presence (Haemaphysalis spinigera), monkey death and human cases in Karnataka, Tamil Nadu, Goa, Maharashtra state of India. **Table S2.** Climatic variables selected for the preliminary and final model. **Table S3.** Correlation matrix among the bio-climatic variables. The Cut-off threshold values are shown (r ≥ 0.8) in bold.

## Data Availability

The datasets of bioclimatic variables analysed during the current study is available in the WorldClim Version2 (http://www.worldclim.com/version2). Further, the datasets generated used and analysed during the current study are available from the corresponding author on reasonable request. All data generated or analysed during this study are included in this published article and further details are available in additional information files.
