# Supplementary material for: Identification of bio-climatic determinants and potential risk areas for Kyasanur forest disease in Southern India using MaxEnt modelling approach
Source: BMC Infect Dis. 2021 Dec 7;21:1226. doi: 10.1186/s12879-021-06908-9 (PMC8650402; doi:10.1186/s12879-021-06908-9)
Supplement: Supplementary file 1 — Additional file 1: Table S1. Showing reported tick presence (Haemaphysalis spinigera), monkey death and human cases in Karnataka, Tamil Nadu, Goa, Maharashtra state of India. Table S2. Climatic variables selected for the preliminary and final model. Table S3. Correlation matrix among the bio-climatic variables. The Cut-off threshold values are shown (r ≥ 0.8) in bold. [file 12879_2021_6908_MOESM1_ESM.doc]

**Additional Table S1:** Showing reported tick presence (Haemaphysalis spinigera), monkey death and human cases in Karnataka, Tamil Nadu, Goa, Maharashtra state of India

| Sl. no | State | District | Locality | Human cases/monkey death/Tick presence | Reported year | Month | References |
| --- | --- | --- | --- | --- | --- | --- | --- |
| 1 | Karnataka  Karnataka | Shimoga | Baragi village | Tick presence | 1957 | December to May | Work & Trapido, 1957 |
| 6 |  | Chamarajanagar | Bandipur National Park | Monkey death | 2013 | November | Mourya et al., 2013 |
| 7 |  | Chamarajanagar | Madduru Forest Range | Human case | 2012 | November | Mourya et al., 2013 |
| 8 |  | Chamarajanagar | Halegoudana camp | Monkey death | 2012 | November | Mourya et al., 2013 |
| 9 |  | Shimoga | Thirthahalli, Hosanagara taluk | Monkey death and Human case | 2014 | January–March | Kiran et al., 2015 |
| 10 |  | Chikmagalur District | - | Human case | 2014 | January–March | Kasabi et al., 2013 |
| 11 |  | Chamarajanagar | - | Tick presence | 2013 | February | Mourya et al., 2013 |
| 12 |  | Chamarajanagar | Madduru Forest Range | Human case | 2013 | January | Mourya et al., 2013 |
| 13 |  | Belgaum | - | Monkey death | 2016 | March 30 –June | Kiran et al., 2015 |
| 14 |  | Shimoga | Thirthahalli Taluk | Human case | 2014 | February | Yadav et al., 2014 |
| 15 |  | Chamarajanagar | Bandipur Tiger Reserve | Monkey death | 2012 | November | Mourya et al., 2013 |
| 16 |  | Shimoga | Kyasanur Forest | Tick presence | 1959 | April 1957 | Trapido, 1959 |
|  |  | Shimoga | - | Tick presence, Monkey death and Human case | 1957-1958 | Mar 1957 & Aug 1958 | Work et al., 1959 |
| 17 |  | Shimoga, Udupi, Mangalore, Chikmagalur and Uttar Kannada | - | Tick presence | 1999 | January, April, Nov | Pattnaik, 2006 |
| 18 |  | Chamarajanagar | - | Tick presence | 2000 | March, April, Dec | Pattnaik, 2006 |
| 19 |  | Shimoga | - | Tick presence | 2001 | Feb-May, July, Dec | Pattnaik, 2006 |
| 20 |  | Shimoga | - | Tick presence | 2002 | Jan-Jun, Aug-Sep, Dec | Pattnaik, 2006 |
| 21 |  | Shimoga, Udupi, Mangalore Chikmagalur, Uttar Kannada | - | Tick presence | 2003 | Jan-May, Oct-Dec | Pattnaik, 2006 |
| 22 |  |  | - | Tick presence | 2004, 2005 | Jan-May, Oct-Dec | Pattnaik, 2006 |
| 24 | Kerala | Wayanad district  Malappuram district | Six villages of Pulpally Community Health Centre (CHC) | Tick presence and Human case | 2014 & 2015 | Dec 2014 & Jun 2015 | Sadanandane et al., 2016 |
| 25 |  |  | Karulai and Amarambalam | Tick presence, and Human case | 2014 & 2015 | Dec 2014 & June 2015 | Sadanandane et al., 2016 |
| 26 |  |  | Nagamala hills in the Nedumkayam Reserve Forest | Tick presence, and Human case | 2014 | 18-22 May | Tandale et al., 2015 |
| 27 | Tamil Nadu | Tiruvannamalai & Vellore District | Amirdi | Tick presence | 1974 | - | Ghalsasi & Dhanda, 1974 |
| 28 |  | Nilgiri | - | Monkey death | 2012 | November | Mourya et al 2013 |
| 29 | Goa | Satara | Pratapgarh | Tick presence | 1974 | - | Ghalsasi & Dhanda, 1974 |
| 30 |  | Kolaba | Poladpur | Tick presence | 1974 | - | Ghalsasi & Dhanda, 1974 |
| 31 |  | Goa | Pali village, Sattari taluk | Human case and monkey death | 2016 | March- June | Patil et al., 2017 |
| 32 | Maharashtra | Pune | Kerala | Tick presence | 1974 | - | Gurav et al., 2016; Ghalsasi & Dhanda, 1974 |
| 33 |  | Pune | Bhimshankar, Pokhari, Gohe, Taleghar, Rajapur | Tick presence | 1974 | - | Gurav et al., 2016; Ghalsasi & Dhanda, 1974 |
| 34 |  | Sindhudurg | Ker, Zolambe, Kolzar and Asaniye village of Dodamarg taluk | Human case | 2016 | January-February 2016 | Awate et al., 2016 |

**Additional Table S2:** Climatic variables selected for the preliminary and final model

| **Code** | **Bio-climatic variable** | **Unit** | **Selected variables for final model** |
| --- | --- | --- | --- |
| **Bio1** | Annual mean temperature | °C | ✓ |
| **Bio2** | Mean diurnal temperature range (mean of monthly (max temp - min temp)) | °C | ✓ |
| **Bio3** | Isothermality ((Bio2/Bio7) *100) |  |  |
| **Bio4** | Temperature seasonality (standard deviation*100) | C of V |  |
| **Bio5** | Maximum temperature of warmest month | °C |  |
| **Bio6** | Minimum temperature of coldest month | °C |  |
| **Bio7** | Temperature annual range (Bio5 - Bio6) | °C |  |
| **Bio8** | Mean temperature of wettest quarter | °C | ✓ |
| **Bio9** | Mean temperature of driest quarter | °C | ✓ |
| **Bio10** | Mean temperature of warmest quarter | °C | ✓ |
| **Bio11** | Mean temperature of coldest quarter | °C | ✓ |
| **Bio12** | Annual precipitation | mm | ✓ |
| **Bio13** | Precipitation of wettest period | mm | ✓ |
| **Bio14** | Precipitation of driest period | mm | ✓ |
| **Bio15** | Precipitation seasonality (CV) | C of V | ✓ |
| **Bio16** | Precipitation of wettest quarter | mm | ✓ |
| **Bio17** | Precipitation of driest quarter | mm | ✓ |
| **Bio18** | Precipitation of warmest quarter | mm | ✓ |
| **Bio19** | Precipitation of coldest quarter | mm | ✓ |

**Additional Table S3:** Correlation matrix among the bio-climatic variables. The Cut-off threshold values are shown (r ≥ 0.8) in bold.

| Variables | Bio1 | Bio2 | Bio3 | Bio4 | Bio5 | Bio6 | Bio7 | Bio8 | Bio9 | Bio10 | Bio11 | Bio12 | Bio13 | Bio14 | Bio15 | Bio16 | Bio17 | Bio18 | Bio19 |
| --- | --- | --- | --- | --- | --- | --- | --- | --- | --- | --- | --- | --- | --- | --- | --- | --- | --- | --- | --- |
| Bio1 | 1 | -0.14 | 0.13 | -0.09 | 0.30 | 0.52 | -0.15 | 0.74 | 0.39 | 0.58 | 0.63 | -0.08 | -0.12 | 0.14 | -0.19 | -0.10 | 0.10 | 0.02 | 0.03 |
| Bio2 | -0.14 | 1 | -0.75 | 0.78 | 0.79 | **-0.83** | **0.91** | 0.27 | -0.04 | 0.57 | -0.66 | -0.55 | -0.43 | -0.48 | 0.47 | -0.40 | -0.49 | -0.52 | -0.47 |
| Bio3 | 0.13 | -0.75 | 1 | **-0.95** | **-0.84** | **0.85** | **-0.94** | -0.39 | 0.03 | -0.69 | 0.79 | 0.25 | 0.14 | 0.32 | -0.72 | 0.08 | 0.29 | 0.32 | 0.38 |
| Bio4 | -0.09 | 0.78 | **-0.95** | 1 | **0.86** | **-0.86** | **0.95** | 0.50 | 0.10 | 0.74 | **-0.82** | -0.32 | -0.22 | -0.24 | 0.67 | -0.16 | -0.21 | -0.37 | -0.35 |
| Bio5 | 0.30 | 0.79 | **-0.84** | **0.86** | 1 | -0.62 | **0.88** | 0.67 | 0.14 | 0.92 | -0.47 | -0.44 | -0.36 | -0.29 | 0.51 | -0.30 | -0.29 | -0.43 | -0.39 |
| Bio6 | 0.52 | **-0.83** | **0.85** | **-0.86** | -0.62 | 1 | **-0.91** | -0.07 | 0.13 | -0.35 | **0.95** | 0.27 | 0.15 | 0.37 | -0.66 | 0.12 | 0.34 | 0.34 | 0.37 |
| Bio7 | -0.15 | 0.91 | **-0.94** | **0.95** | **0.88** | **-0.91** | 1 | 0.39 | -0.01 | 0.69 | -0.78 | -0.39 | -0.28 | -0.37 | 0.66 | -0.22 | -0.35 | -0.42 | -0.42 |
| Bio8 | 0.74 | 0.27 | -0.39 | 0.50 | 0.67 | -0.07 | 0.39 | 1 | 0.46 | **0.86** | 0.01 | -0.15 | -0.13 | 0.01 | 0.26 | -0.08 | -0.01 | -0.07 | -0.15 |
| Bio9 | 0.39 | -0.04 | 0.03 | 0.10 | 0.14 | 0.13 | -0.01 | 0.46 | 1 | 0.30 | 0.13 | -0.05 | -0.05 | 0.01 | 0.02 | -0.04 | 0.02 | -0.12 | 0.09 |
| Bio10 | 0.58 | 0.57 | -0.69 | 0.74 | **0.92** | -0.35 | 0.69 | **0.86** | 0.30 | 1 | -0.22 | -0.31 | -0.26 | -0.13 | 0.43 | -0.19 | -0.14 | -0.29 | -0.27 |
| Bio11 | 0.63 | -0.66 | 0.79 | **-0.82** | -0.47 | **0.95** | -0.78 | 0.01 | 0.13 | -0.22 | 1 | 0.20 | 0.11 | 0.24 | -0.61 | 0.07 | 0.19 | 0.28 | 0.30 |
| Bio12 | -0.08 | -0.55 | 0.25 | -0.32 | -0.44 | 0.27 | -0.39 | -0.15 | -0.05 | -0.31 | 0.20 | 1 | **0.96** | 0.05 | 0.29 | **0.97** | 0.11 | 0.67 | 0.64 |
| Bio13 | -0.12 | -0.43 | 0.14 | -0.22 | -0.36 | 0.15 | -0.28 | -0.13 | -0.05 | -0.26 | 0.11 | **0.96** | 1 | -0.10 | 0.44 | **0.99** | -0.06 | 0.59 | 0.56 |
| Bio14 | 0.14 | -0.48 | 0.32 | -0.24 | -0.29 | 0.37 | -0.37 | 0.01 | 0.01 | -0.13 | 0.24 | 0.05 | -0.10 | 1 | -0.46 | -0.08 | **0.95** | 0.20 | 0.12 |
| Bio15 | -0.19 | 0.47 | -0.72 | 0.67 | 0.51 | -0.66 | 0.66 | 0.26 | 0.02 | 0.43 | -0.61 | 0.29 | 0.44 | -0.46 | 1 | 0.48 | -0.43 | 0.01 | -0.06 |
| Bio16 | -0.10 | -0.40 | 0.08 | -0.16 | -0.3 | 0.12 | -0.22 | -0.08 | -0.04 | -0.19 | 0.07 | **0.97** | **0.99** | -0.08 | 0.48 | 1 | -0.04 | 0.61 | 0.56 |
| Bio17 | 0.10 | -0.49 | 0.29 | -0.21 | -0.29 | 0.34 | -0.35 | -0.01 | 0.02 | -0.14 | 0.19 | 0.11 | -0.06 | **0.95** | -0.43 | -0.04 | 1 | 0.22 | 0.17 |
| Bio18 | 0.02 | -0.52 | 0.32 | -0.37 | -0.43 | 0.34 | -0.42 | -0.07 | -0.12 | -0.29 | 0.28 | 0.67 | 0.59 | 0.20 | 0.01 | 0.61 | 0.22 | 1 | 0.24 |
| Bio19 | 0.03 | -0.47 | 0.38 | -0.35 | -0.39 | 0.37 | -0.42 | -0.15 | 0.09 | -0.27 | 0.30 | 0.64 | 0.56 | 0.12 | -0.06 | 0.56 | 0.17 | 0.24 | 1 |
